# The Determination of HIV-1 RT Mutation Rate, Its Possible Allosteric Effects, and Its Implications on Drug Resistance

**DOI:** 10.3390/v12030297

**Published:** 2020-03-09

**Authors:** Joshua Yi Yeo, Ghin-Ray Goh, Chinh Tran-To Su, Samuel Ken-En Gan

**Affiliations:** 1 Antibody & Product Development Lab, Bioinformatics Institute, Agency for Science, Technology and Research (A*STAR), Singapore 138671, Singapore; yeoyj@bii.a-star.edu.sg (J.Y.Y.); gohgr@intern.bii.a-star.edu.sg (G.-R.G.); chinhsutranto@bii.a-star.edu.sg (C.T.-T.S.); 2 p53 Laboratory, Agency for Science, Technology and Research (A*STAR), Singapore 138648, Singapore; 3 Experimental Drug Development Centre, Agency for Science, Technology and Research (A*STAR), Singapore 138670, Singapore

**Keywords:** retroviruses, HIV-1, reverse transcriptase, mutation rate, drug resistance, allostery

## Abstract

The high mutation rate of the human immunodeficiency virus type 1 (HIV-1) plays a major role in treatment resistance, from the development of vaccines to therapeutic drugs. In addressing the crux of the issue, various attempts to estimate the mutation rate of HIV-1 resulted in a large range of 10^−5^–10^−3^ errors/bp/cycle due to the use of different types of investigation methods. In this review, we discuss the different assay methods, their findings on the mutation rates of HIV-1 and how the locations of mutations can be further analyzed for their allosteric effects to allow for new inhibitor designs. Given that HIV is one of the fastest mutating viruses, it serves as a good model for the comprehensive study of viral mutations that can give rise to a more horizontal understanding towards overall viral drug resistance as well as emerging viral diseases.

## 1. Introduction

Among the retroviruses, HIV-1 is the most genetically diverse [1,2,3,4], due to its higher mutation rate (reviewed by [5,6]), genetic recombination, and fast viral replication [7,8,9], where multiple unique sequences can be isolated from within single patients [10,11,12,13,14]. This diversity allows for the emergence of resistance strains, and evasion from the host immune system and drug therapies. Under selection pressures (such as antiretroviral drugs), these drug-resistant mutations accumulate and become dominant strains [7,15], rendering treatment regimens ineffective and hindering the development of effective vaccines [16]. Thus, to combat viral diseases, HIV is an ideal model for studying drug resistance and emerging infections.

The genetic recombination of two single drug resistant strains can give rise to HIV-1 virions with multiple drug resistance [17,18,19,20]. Coupled with high mutation rates, the estimated production of 10^9^ virions per day within an infected individual results in numerous mutant variants in a few viral generations [21], with drug-resistant strains completely replaced by wild type strains within 2–4 weeks after antiretroviral treatment [7]. 

The error rates and mutation frequencies of HIV-1 reverse transcriptase (RT) are reported to be approximately 10^−5^–10^−3^ errors/bp/cycle and 10^−4^–10^−2^ mutants/clones, respectively. However, the lack of consensus in mutation rates observed in HIV-1 suggests that multiple factors and contributors are involved (see Table 1 and Table 3).

In this review, we discuss the various experimental fidelity assays, their reported mutation rates, and the potential of non-active site allosteric mutations. 

## 2. HIV-1 Reverse Transcriptase (RT)

**Figure 1:**
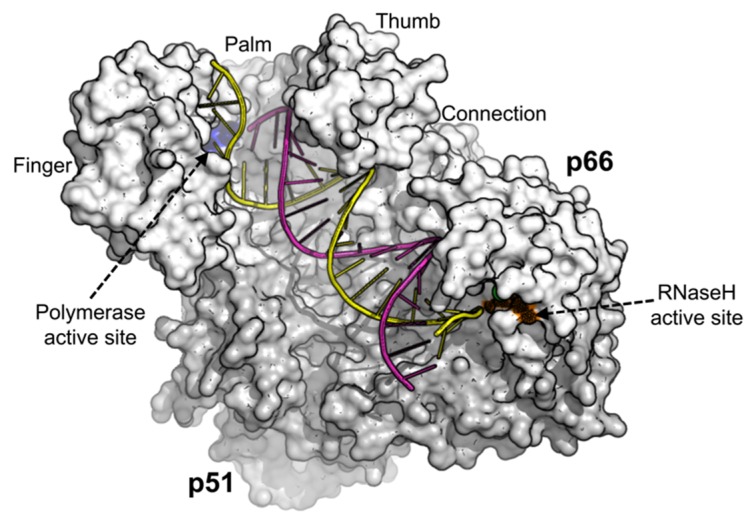
Human immunodeficiency virus type 1 (HIV-1) Reverse Transcriptase structure complexed with DNA (pdb 1T05) [27]. The image was generated using PyMOL [28].

HIV-1 RT is an asymmetric heterodimer consisting of the p66 (66 kDa) and the p51 (51 kDa) subunits [22] illustrated in Figure 1. It lacks the 3′ exonuclease proofreading activity [23], contributing up to 68% mutations in cell-based assays during early stage replication (minus-strand synthesis and RNA transcription) and 32% during late stage replication (plus-strand synthesis and DNA repair) [24]. HIV-1 RT has been found to contribute to HIV-1 mutagenesis [25], accounting for 59.7% of the mutations in the viral RNA and 2.0% in the viral DNA [26].

## 3. Comparison of Cell-Free and Cell-Based Fidelity Assays

The fidelity of HIV-1 RT is best assessed directly on patient samples, but ethical and biosafety requirements are insurmountable obstacles for many small labs/institutes. Even without such obstacles, the virus would have undergone multiple generations of replication within a patient between sampling, making it nearly impossible to determine the fidelity of a single round of replication. Thus, various cell-free and cell-based approaches were developed.

### 3.1. Cell-Free Fidelity Assays

In vitro assays, termed “cell-free” assays to emphasize the absence of HIV replication in cells, reduce confounding factors (e.g., a balanced dNTP pool) to give higher reproducibility. However, such reductionist methods also remove factors that influence the fidelity of HIV RT, while employing high temperatures in PCR that could disrupt secondary structures of the template nucleic acid. 

The first cell-free fidelity assays utilized synthetic polynucleotide templates to determine errors using a mismatched radio-labelled nucleotide [29]. However, these homopolymer templates do not accurately represent the natural heteropolymer template (such as a gene), resulting in the overestimation of mutation rates due to their repeating nature [30]. Confounding factors such as the slippage of primers and stacking interactions between nucleotides further influence the fidelity of base substitution [29]. 

For the visual identification of mutants and non-mutants, reporter genes such as the α-complementing region of the lacZ gene (lacZα) and the DNA of bacteriophage ΦX174 are commonly employed in base reversion assays that measure the error rate of RT on a single base [30]. However, RNA secondary structure influences are not considered, overlooking the spectrum of mutations and any potential mutational hotspots. To address this limitation, the cell-free forward mutation assay [31] involving gap filling by RT, allows the determination of mutation spectra and hotspots. The error rates and mutant frequencies of HIV-1 RT from previously reported cell-free fidelity assays are shown in Table 1 and Table 2, respectively.

### 3.2. Cell-Based Fidelity Assays

In vivo assays, termed “cell-based” assays here, attempt to mimic the conditions to include host and viral proteins absent in cell-free assays. However, this trades off reproducibility when different cells are utilized. The assays leverage on transfecting shuttle vectors containing HIV genes into mammalian cells, followed by the selection of mutants in suitable hosts (e.g., bacteria). However, silent mutations are not detected, possibly leading to an overestimated fidelity of HIV RT in both cell-based and cell-free assays. The error rates and mutant frequencies of HIV-1 RT from previously reported cell-based fidelity assays are shown in Table 3 and Table 4, respectively.

## 4. Studies on HIV-1 Genes

For a better study of HIV-1 RT, the mutation positions are important to provide mechanistic insights to the development of drug resistance [76]. The well-established mutation rate of HIV-1 RT is performed on lacZα [39,40,41,42,43,44,45,48,58,77], resulting in a gap of knowledge regarding how HIV-1 genes are mutated by RT.

We found only two studies that utilized HIV templates [36,74]. One involved the HIV-1 *env* gene (see Table 5) in cell-free assays, finding an error rate in the DNA of 1.90 × 10^−4^ errors/bp/cycle, in RNA at 2.00 × 10^−4^ errors/bp/cycle and RNA/DNA at 3.80 × 10^−4^ errors/bp/cycle. This is comparable to previous M13mp2 forward assays using the lacZα template in DNA at 1.69 × 10^−4^ and RNA at 1.45 × 10^−4^ error/bp/cycle [36]. Similarly, Geller and colleagues’ work on HIV *env* and *int-vir-vpr* RNA, found error rates of 0.36 × 10^−4^ and 0.75 × 10^−4^ error/bp/cycle, respectively [74]. 

Mutations reported in the first study were found to partially correlate with those found in AIDS patients [36], while the latter showed that sequence and secondary structure affected the activity of cytidine deamination and fidelity of HIV RT [74]. Together, they demonstrate the need to perform studies directly on HIV-1 genes.

## 5. Analysis of Allosteric Communication 

Current anti-HIV drugs target the key viral enzymes such as reverse transcriptase, integrase, and protease [78], and, as such, occurrences of mutations hotspots within these targets are essential for understanding drug resistance and the design of new drugs.

Clinical mutations were found not only at the direct drug-binding sites but also non-functional regions [79], the latter of which can have indirect effects on drug resistance such as allosteric communication to the catalytic sites. The inhibition of such communication can be explored for novel classes of drugs, such as in the case of reverse transcriptase inhibitors (RTIs) [80], integrase inhibitors [81], and protease inhibitors (PIs) [82]. The non-nucleoside RTIs bind non-competitively to an allosteric site on p66 subunit to cause structural changes in the RT polymerase active site, hindering DNA polymerization. Our ongoing study has found further potential interferences targeting the p51 to affect overall RT activity [83]. On integrase, allosteric inhibitors impair the binding of integrase and the cellular cofactor LEDGF/p75 during HIV-1 replication to induce aberrant integrase multimerization [81]. Similarly, a potential allosteric protease inhibitor was found to bind to a site at the protease flap to equipotently inhibit both wild-type and certain drug-resistant variants [82].

Given the resource constraints for experimentally testing every residue for allosteric communication, *in silico* screening can be performed on potential mutation hotspots. Following experimental validation via recombinant methods to establish the level and type of effect (detrimental or augmented catalytic activity), the site can then be targeted for intervention, especially if they are able to elicit drastic protein structural changes to affect functionality. One such example is in IgA1 and IgA2, which had varying allosteric communications due to different intermediate protein regions [84]. 

Recent advances have shown the p51 subunit of HIV-1 RT to induce flexibility on the DNA polymerase active site on p66, inhibiting RT function [85]. Similarly, Gag non-cleavage site mutations that are known to compensate for viral fitness in drug resistance were found to have allosteric communications [86] with the first protease cleavage site on Gag [87]. While further research is on-going, the example of non-cleavage Gag mutations involved in protease inhibitor resistance (despite an absence of mutations in the cleavage sites) demonstrates the importance of non-active site mutations that exhibit allosteric communications [75,88].

**Figure 2:**
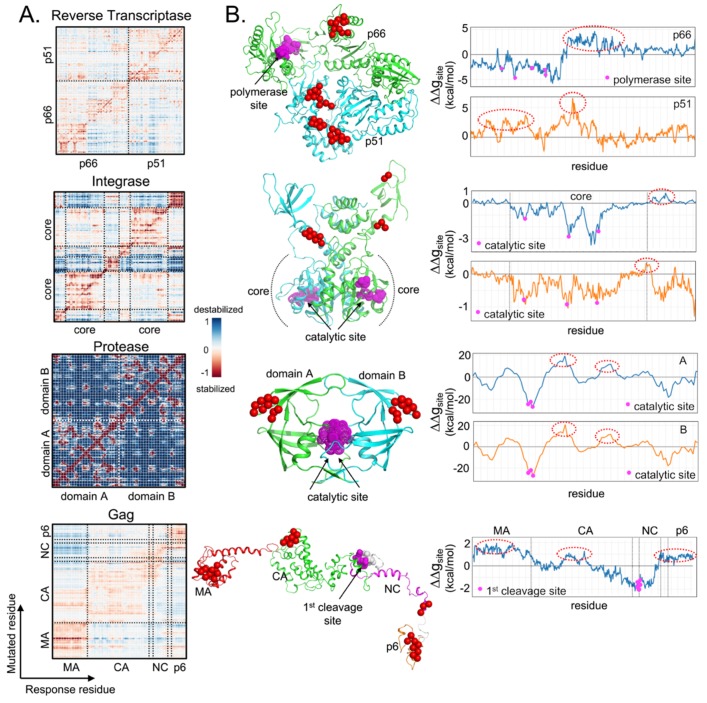
Underlying allosteric communications were found within HIV-1 proteins. (**A**) Allosteric signaling maps (single-point mutation screening) of HIV-1 Reverse Transcriptase, Integrase, Protease, and Gag. Structural presentation using RT (pdb 3T19), IN (reconstructed from pdb 1K6Y and 1EX4), PR (pdb 2PC0), and Gag model from Su et al. [87]; (**B**) Allosteric-free energies (ΔΔg_site_) on specific catalytic or cleavage sites (magenta spheres) were estimated based on individual perturbations at single residues (x-axis) to demonstrate the resulting stabilizing (ΔΔg_site_ < 0) or destabilizing (ΔΔg_site_ > 0) effects. The possible mutations which may potentially destabilize the sites of interest, are highlighted in red spheres and red dash ovals.

For this review, the locations in the various HIV target proteins were screened for their allosteric communication to the known active sites. To quantify the strength of allosteric effects caused by each residual mutation, allosteric-free energy (ΔΔg_residue_) was calculated (using AlloSigMA, more details in [89,90,91]) for the other responding residues. Individual perturbations for the whole protein are shown in the allosteric signaling map [92,93] of Figure 2.

Asymmetrical effects in the HIV-1 RT structure further affirmed that mutations in p51 could stabilize the active site on p66, but not vice versa (Figure 2A). Since rigidity reduces HIV-1 RT activity [94], this opens up p51 as a potential new drug target. Given the estimated allosteric-free energies at the DNA polymerase active site (ΔΔg_site_ by averaging all ΔΔg_residue_ of the residues involving in the active site to demonstrate stabilizing (ΔΔg_site_ < 0) or destabilizing (ΔΔg_site_ > 0) effects), it showed that the active site is destabilized by mutations on the thumb domain of p66 (residues 260-321) and on p51 (residues 33–42, 68–78, and 96–114), highlighted in red spheres and red dash ovals in Figure 2B. These sites can thus be targeted for intervention. 

Differing from RT, symmetrical allosteric communications between domains in HIV-1 integrase and protease are found [95,96], probably resulting from the homo-multimerization (Figure 2A). Mutations on the C-terminal domain (CTD, residues 220–221, 230–232, and 211–217) of integrase and at the “ears” regions of protease (residues 33–45 and 57–63, of which residues L33, E34, and M36 were reported to be resistant to several protease inhibitors [79]) have been found to affect the active sites of the two enzymes (Figure 2B). For the Gag protein, allosteric communications between several non-cleavage sites and the cleavage sites were found, affecting proteolysis [87], e.g., on Gag matrix (MA: residues E12, V35, E40, and L75), capsid (CA: residue H219), and p6 (L449 and P453) domains [88,97,98]. By analyzing mutational hotspots, additional sites with allosteric communications can be identified for pre-emptive drug design.

## 6. Implications on Sagacious Drug Design

The control of viral mutation rates has been proposed as an antiretroviral strategy [6]. In this review, we further propose identifying mutational hotspots and mutation rates of specific viral drug targets towards an application of rational drug design, that can include the development and pre-emptive design of novel drugs. While generic HIV-1 RT mutation rates are well-established from multiple studies reviewed in this article, there are still gaps when looking at specific HIV genes, especially given that sequence context and secondary structure are known to influence the fidelity of HIV-1 RT [74]. Within these targets, an in-depth understanding of the mutation rate and types of mutation (e.g., substitution, deletion and insertion) and predisposition to specific nucleotide changes (e.g., A to G) can shed light on viral drug resistance. Such knowledge allows for the targeting of regions with lower mutation rates while balancing possible allosteric effects or compensatory effects of viral fitness in a sagacious drug design strategy. For example, the M184V resistance mutation in HIV-1 RT is known to increase fidelity, impair viral fitness, and increase hyper-sensitization to NRTIs (such as amprenavir and efavirenz) [99]. Thus, it is possible to leverage on such features alongside structural understanding [76] to utilize combinatorial therapies to target the active site, using existing inhibitors to select for the mutation alongside other inhibitors to limit the escape mutations. 

## 7. Conclusions and Future Perspective

Comparing cell-free and cell-based essays that contribute to the mutation rate of HIV-1, there is a lack of study of specific HIV-1 drug targets that would provide insights for rational drug designs, especially in the light of in silico allosteric analysis. Addressing such gaps has great promise for gaining an upper hand to develop novel intervention strategies against HIV.

## Figures and Tables

**Table 1:** Error rates of HIV-1 reverse transcriptase (RT) measured in cell-free fidelity assays.

Assay	RT Mutant	Vector	Reporter Gene/Template	Error Rate (× 10^−4^ errors/bp/cycle)	Reference
Base reversion	WT ^1^	ΦX174 am3	Position 587 of ΦX174 am3, DNA	2.50	[32]
Base reversion	WT ^1^	M13mp2	Position 89 of lacZα coding sequence, DNA	0.55	[23]
Forward mutation			*lacZ*α, DNA	5.00–6.67	
Misincorporation	WT (HTLV-III_B_)	-	Polyadenylic acid	0.31–0.57	[33]
	WT (HIV[GUN-1])	-		0.26–0.38	
Base reversion	WT (HTLV-III)	ΦX174 am16	ΦX174 am16, DNA	1.43–2.00	[34]
Forward mutation	WT ^1^	-	*lacZ*α, RNA	1.45	[35]
		-	M13mp2 (+), DNA	1.69	
Forward mutation	WT ^1^	-	*env* V-1, DNA	1.90	[36]
		-	*env* V-1, RNA	2.00	
		-	*env* V-1, RNA/DNA	3.80	
Forward mutation	WT (BH10)	M13mp19 (CSIVM13B)	SIV_agm_TYO-7 *env* (minus-strand)*,* DNA	0.18	[37]
		M13mp19 (CSIVM13B)	*lacZ*α, DNA	0.53	
Forward mutation	WT (BH10)	M13mp2	*lacZ*α, DNA	0.45	[38]
	Q151M (BH10)			0.40	
	A62V/V75I/F77L/F116Y/Q151M (BH10)			0.23	
Forward mutation	WT (HXB2)	M13mp2	*lacZ*α, DNA	0.57	[39]
	E89G (HXB2)			0.41	
	M184V (HXB2)			0.36	
	E89G/M184V (HXB2)			0.81	
Forward mutation	WT (HXB2)	M13mp2	*lacZ*α, DNA	0.71	[39,40]
	M184V (HXB2)			0.43	
Forward mutation	M184I (HXB2)	M13mp2	*lacZ*α, DNA	0.17	[40]
Forward mutation	WT ^1^	U-DNA Litmus 29 (Not)	*lacZ*α, DNA	1.60	[41]
	Y115F ^1^			1.00	
	Y115V ^1^			4.70	
Forward mutation	WT (HXB2)	M13mp2	*lacZ*α, DNA	0.57	[42]
	K65R (HXB2)			0.07	
	L74V (HXB2)			0.30	
Forward mutation	WT (NL4-3)	M13mp2	*lacZ*α, DNA	0.63	[43]
	FE20 (NL4-3)			0.56	
	FE103 (NL4-3)			0.53	
Forward mutation	WT ^1^	U-DNA Litmus 29 (Not)	*lacZ*α, DNA	0.75	[44]
Forward mutation	WT (BH10)	M13mp2	*lacZ*α, DNA	1.36	[45]
	WT (ESP49)			0.55	
	V75I (ESP49)			0.29	
Forward mutation	D433N (ESP49)	M13mp2	*lacZ*α, DNA	0.14	[46]
	E478Q (ESP49)			0.1	
	V75I/D443N (ESP49)			0.14	
	V75I/E478Q (ESP49)			0.2	
	E478Q (BH10)			0.42	
Forward mutation	WT (BH10)	M13mp2	*lacZ*α, DNA	1.52	[47]
	WT (ROD)			1.00	
	K65R (ROD)			0.84	
	K65R/Q151M/M184V (ROD)			0.74	
Forward mutation	WT (BH10)	M13mp2	*lacZ*α, RNA	0.35	[48]
	WT (ESP49)			0.27	
	K65R (ESP49)			0.26	
	K65R/V75I (ESP49)			0.25	

^1^ Lab strain of HIV-1 RT used was not mentioned.

**Table 2:** Mutant frequencies of HIV-1 RT measured in cell-free fidelity assays.

Assay	RT Mutant	Vector	Reporter Gene/Template	Mutant Frequency (× 10^−4^ Mutants/Clones)	Reference
Base reversion	WT ^1^	M13mp2	Position 89 of *lacZ*α coding sequence, DNA	1.00	[23]
Forward mutation	WT ^1^	M13mp2	*lacZ*α, DNA	340–460	
Forward mutation	WT ^1^	M13mp2	*lacZ*α, DNA	390.00	[49]
Base reversion	WT ^1^	pTZ18R	ΦX174 am16, RNA	26.00	[50]
			ΦX174 am16, DNA	26.00	
Forward mutation	WT ^1^	M13mp2	*lacZ*α, DNA	340–540	[51]
			*lacZ*α, RNA	91–210	
Forward mutation	WT ^1^	M13mp2	*lacZ*α, RNA	40.70	[35]
		pBluescript SK+	M13mp2, (+) DNA	47.30	
Forward mutation	WT ^1^	M13mp2	pseudowild type 1 (pwt1) *lacZα*, DNA	490	[52]
			pseudowild type 2 (pwt2) *lacZα*, DNA	450	
			*lacZ*α, DNA	500	
Forward mutation	WT (HXB2)	M13mp2	*lacZ*α, DNA	200	[53]
	D256A (HXB2)			240	
	Q258A (HXB2)			390	
	K259A (HXB2)			300	
	L260A (HXB2)			230	
	G262A (HXB2)			880	
	K263A (HXB2)			290	
	W266A (HXB2)			640	
	Q269A (HXB2)			510	
Forward mutation	WT (HXB2)	M13mp2	*lacZ*α, DNA	210	[54]
	G262A (HXB2)			860	
	W266A (HXB2)			630	
Forward mutation	WT (HXB2)	M13mp2	*lacZ*α, DNA	160	[55]
	R277A (HXB2)			140	
	Q278A (HXB2)			190	
	L279A (HXB2)			150	
	C280A (HXB2)			300	
	K281A (HXB2)			140	
	L282A (HXB2)			120	
	R284A (HXB2)			170	
	G285A (HXB2)			160	
	K287A (HXB2)			120	
Forward mutation	WT (BH10)	M13mp19 (CSIVM13B)	SIV_agm_TYO-7 *env* (minus-strand)*,* DNA	31.40	[37]
			*lacZ*α, DNA	60.90	
Forward mutation	WT (BH10)	M13mp2	*lacZ*α, DNA	232	[56]
	D76V (BH10)			26	
Forward mutation	WT (BH10)	M13mp2	*lacZ*α, DNA	64.00	[38]
	Q151M (BH10)			55.00	
	A62V/V75I/F77L/F116Y/Q151M (BH10)			31.00	
Forward mutation	WT (HXB2)	M13mp2	*lacZ*α, DNA	86.00	[39]
	E89G (HXB2)			62.60	
	M184V (HXB2)			55.30	
	E89G/M184V (HXB2)			123.00	
Forward mutation	WT (HXB2)	M13mp2	*lacZ*α, DNA	97	[39,40]
	M184V (HXB2)			59	
Forward mutation	M184I (HXB2)	M13mp2	*lacZ*α, DNA	24	[40]
Forward mutation	WT (BH10)	M13mp2	*lacZ*α, DNA	250	[57]
	R78A (BH10)			28	
Base reversion	WT (HXB2)		TGA codon (position 87–89) in lacZα, DNA	2.2	[58]
	R72A (HXB2)			82	
One-nucleotide deletion reversion	WT (HXB2)		TTTT run in M13mp2 DNA	32	
	R72A (HXB2)			1.6	
Forward mutation	WT (HXB2)		*lacZ*α, DNA	210	
	R72A (HXB2)			340	
Forward mutation	WT ^1^	U-DNA Litmus 29 (Not)	*lacZ*α, DNA	278.00	[41]
	Y115F ^1^			175.00	
	Y115V ^1^			82.00	
Forward mutation	WT (BH10)	M13mp2	*lacZ*α, DNA	192	[59]
	L74V (BH10)			55	
	E89G (BH10)			96	
	M184V (BH10)			228	
	Y183F (BH10)			303	
	Y115A (BH10)			763	
Forward mutation	WT (HXB2)	M13mp2	*lacZ*α, DNA	86	[42]
	K65R (HXB2)			10.6	
	L74V (HXB2)			50.5	
Forward mutation	WT (BH10)	M13mp2	*lacZ*α, DNA	261	[60]
	Q151N (BH10)			20	
	K154A (BH10)			125	
Forward mutation	WT (NL4-3)	M13mp2	*lacZ*α, DNA	86.00	[43]
	FE20 (NL4-3)			77.00	
	FE103 (NL4-3)			74.00	
Forward mutation	WT (HXB2)	M13mp2	*lacZ*α, DNA	97	[61]
	F61A (HXB2)			8.3	
Forward mutation	V184I (HXB2)	M13mp2	*lacZ*α, DNA	30	[62]
Forward mutation	E89K (HXB2)	M13mp2	*lacZ*α, DNA	77	[63]
	E89V (HXB2)			64	
	E89S (HXB2)			53	
Forward mutation	WT (HXB2)	M13mp2	*lacZ*α, DNA	97	[64]
	T69S-AG (HXB2)			20	
	T69S-SG (HXB2)			12	
	T69S-SS (HXB2)			24	
	A62V/T69S-AG/L210W/R211K/L214F/T215Y			8.5	
	A62V/T69S-SG/L210W/R211K/L214F/T215Y			19	
	A62V/T69S-SS/L210W/R211K/L214F/T215Y			11	
	M41L/T69S-AG/L210W/R211K/L214F/T215Y			6.3	
	M41L/T69S-SG/L210W/R211K/L214F/T215Y			5.9	
Forward mutation	WT ^1^	U-DNA Litmus 29 (Not)	*lacZ*α, DNA	130.00	[44]
Forward mutation	WT (BH10)	M13mp2	*lacZ*α, DNA	206	[65]
	V75A (BH10)			281	
	V75F (BH10)			112	
	V75I (BH10)			69.6	
Base reversion	WT (BH10)			27	
	V75I (BH10)			7.8	
Forward mutation	WT (ESP49)	M13mp2	*lacZ*α, DNA	83.1	[45]
	V75I (ESP49)			43.4	
Forward mutation	K65R (ESP49)	M13mp2	*lacZ*α, DNA	7.7	[66]
	K65R/V75I (ESP49)			8.9	
	R78A (ESP49)			5.9	
Forward mutation	WT (ESP49)	M13mp2	*lacZ*α, DNA	96	[46]
	D433N (ESP49)			19.8	
	E478Q (ESP49)			13.5	
	V75I/D443N (ESP49)			18.2	
	V75I/E478Q (ESP49)			29.1	
	WT (BH10)			113.4–132.3	
	E478Q (BH10)			57.6	
Forward mutation	WT (BH10)	M13mp2	*lacZ*α, DNA	199.00	[47]
	WT (ROD)			124.20	
	K65R (ROD)			117.90	
	K65R/Q151M/M184V (ROD)			103.10	
Forward mutation	WT (BH10)	M13mp2	*lacZ*α, DNA	40.50	[48]
	WT (ESP49)			34.5	
	K65R (ESP49)			29.5	
	K65R/V75I (ESP49)			29.3	

^1^ The lab strain of HIV-1 RT used was not mentioned.

**Table 3:** Error rates of HIV-1 RT measured in cell-based fidelity assays.

Assay	RT Mutant	Vector	Reporter Gene/Template	Error Rate (× 10^−4^ errors/bp/cycle)	Reference
Forward mutation	WT (NL4-3)	HIV-1 vector (HIV shuttle 3.12 & 5.1)	*lacZ*α, DNA	0.34	[67]
Forward mutation	WT (NL4-3)	HIV-1 vector (HIV shuttle 3.12)	*lacZ*α, DNA	0.40	[68]
Forward mutation	WT (NL4-3)	HIV-1 vector (HIV shuttle 3.12 *vpr^+^)*	*lacZ*α, DNA	0.30	[69]
		HIV-1 vector (HIV shuttle 3.12 *vpr* ATG^−^)		1.20	
Forward mutation (SSCP)	WT (HXB2)	HIV-1 vector (pHIV-gpt)	HIV-1 LTR, DNA	0.92	[25]
	WT (NL4-3)	HIV-1 vector (NL4-3gpt)		0.79	
Forward mutation	WT (NL4-3)	HIV-1 vector (pNL4-3deltaΔ +cass)	*tk,* DNA	0.22	[70]
Forward mutation	WT (NL4-3)	HIV-1 vector (pNL4-3 HIG)	U373-MAGI-X4 cells, DNA	6.90	[71]
Forward mutation	WT (NL4-3)	HIV-1 vector (pSICO-LZF)	*lacZ*α, DNA	0.22	[72]
		HIV-1 vector (pSICO-LZR)		0.17	
Forward mutation	WT (NL4-3)	HIV-1 vector (pSICO-LZF/R)	*lacZ*α, DNA	0.14	[73]
	Y115F (NL4-3)			0.37	
	Q151M (NL4-3)			0.17	
	M184I (NL4-3)			0.21	
	M184V (NL4-3)			0.18	
Forward mutation	WT (HX2B2)	HIV-1 vector (pSDY-dCK)	HIV *env*, RNA	0.36	[74]
			HIV *Int-vif-vpr*, RNA	0.75	

**Table 4:** Mutant frequencies of HIV-1 RT measured in cell-based fidelity assays.

Assay	RT Mutant	Vector	Reporter Gene/Template	Mutant Frequency (× 10^−4^ Mutants/Clones)	Reference
Forward mutation	WT (NL4-3)	HIV-1 vector (HIV shuttle 3.12)	*lacZ*α, DNA	44	[67]
		HIV-1 vector (HIV shuttle 5.2)		42	
Forward mutation	WT (NL4-3)	HIV-1 vector (HIV shuttle 3.12)	*lacZ*α, DNA	50	[68]
Forward mutation	WT (NL4-3)	HIV-1 vector (HIV shuttle 3.12 *vpr* ATG^−^)	*lacZ*α, DNA	150	[69]
		HIV-1 vector (HIV shuttle 3.12 *vpr* A30F)		140	
		HIV-1 vector (HIV shuttle 3.12 *vpr^+^)*		40	
Forward mutation	WT (NL4-3)	HIV-1 vector	*lacZ*α, DNA	1500–1510 ^2^	[75]
	K65R (NL4-3)			450 ^2^	
	D67N (NL4-3)			1490 ^2^	
	K70R (NL4-3)			1470^2^	
	L74V (NL4-3)			1120^2^	
	D76V (NL4-3)			590–600 ^2^	
	R78A (NL4-3)			420–430 ^2^	
	E89G (NL4-3)			120^2^	
	Y115A (NL4-3)			3400–3480 ^2^	
	Q151N (NL4-3)			250–280 ^2^	
	K154A (NL4-3)			1520 ^2^	
	F227A (NL4-3)			930 ^2^	
	W229A (NL4-3)			720 ^2^	
	Y501W (NL4-3)			4300 ^2^	
	I505A (NL4-3)			1410 ^2^	
	D76V/R78A (NL4-3)			150 ^2^	
	R78A/Q151N (NL4-3)			110 ^2^	
	Y115A/Q151N (NL4-3)			1050 ^2^	
Forward mutation	WT (NL4-3)	HIV-1 vector (HIV shuttle 3.12)	*lacZ*α, DNA	1490	[62]
	V148I (NL4-3)			390	
	Q151N (NL4-3)			260	
Forward mutation	WT (NL4-3)	HIV-1 vector (pSICO-LZF/R)	*lacZ*α (F), DNA	38	[72]
		HIV-1 vector (pSICO-LZF/R)	*lacZ*α (R), DNA	21.8	
		HIV-1 vector (pNLZeoIN-R-E-.LZF/R)	*lacZ*α (F), DNA	21.7	
		HIV-1 vector (pNLZeoIN-R-E-.LZF/R)	*lacZ*α (R), DNA	18.2	
Forward mutation	WT (NL4-3)	HIV-1 vector (pSICO-LZF/R)	*lacZ*α, DNA	21.98	[73]
	Y115F (NL4-3)			55.91	
	Q151M (NL4-3)			25.69	
	M184I (NL4-3)			31.9	
	M184V (NL4-3)			27	

^2^ Mutant frequency was calculated as mutants/cycle.

**Table 5:** Percentages of nucleotide mutations of HIV-1 RT on the HIV-1 gene and LacZα template.

Template	Base Substitutions												Frameshifts		Others	Reference
	Transversions								Transitions				Insertions	Deletions		
	A -> C	C -> A	A -> T	T -> A	C -> G	G -> C	G -> T	T -> G	G -> A	A -> G	C -> T	T -> C				
HIV-1 *env* V-1, DNA	0	0	1.59 (1)	1.59 (1)	1.59 (1)	0	4.76 (3)	6.35 (4)	9.52 (6)	26.98 (17)	15.87 (10)	4.76 (3)	23.81 (15)	3.17 (2)	0	[36]
HIV-1 *env* V-1, DNA/RNA	7.55 (4)	0	3.77 (2)	3.77 (2)	0	0	16.98 (9)	0	3.77 (2)	20.75 (11)	16.98 (9)	13.21 (7)	7.55 (4)	5.66 (3)	0	
HIV-1 *env*	27.88 (29)								46.15 (48)	15.38 (16)			8.65 (9)	1.92 (2)	0	[74]
HIV-1 *int-vif-vpr*	19.61 (20)								50.98 (52)	22.55 (23)			4.90 (2)	1.96 (2)	0	
*LacZα*, RNA	-	-	-	-	-	10.64 (5)	23.40 (11)	-	0	-	31.91 (15)	2.13 (1)	14.89 (7)		17.02 (8)	[35]
LacZα, DNA	0	31.53 (70)	0	30.63 (68)	0.45 (1)	0.45 (1)	0	0.90 (2)	12.61 (28)	3.60 (8)	4.95 (11)	0	14.86 (33)		0	[41]
LacZα, DNA	0.57 (1)	11.93 (21)	0	9.66 (17)	0	2.27 (4)	0	0.57 (1)	45.45 (80)	6.25 (11)	7.39 (13)	1.14 (2)	10.23 (18)		4.55 (8)	[44]

Reported percentages were calculated using previous research, with exact reported numbers indicated in parentheses.
